# Identifying ubiquitinated proteins and aggregates

**DOI:** 10.18632/aging.101605

**Published:** 2018-10-18

**Authors:** Nathan Basisty, Birgit Schilling, Peter S. Rabinovitch

**Affiliations:** 1 The Buck Institute for Research on Aging, Novato, CA 94945, USA; 2 Department of Pathology, University of Washington, Seattle, WA 98105, USA

**Keywords:** protein aggregates, ubiquitination, mass spectroscopy, proteostasis, aging

The accumulation of intra- and extracellular inclusions and protein aggregates are well-known hallmarks of aging that are associated with the incidence and progression of diseases of multiple tissues. While the pathology and structure of some varieties of inclusions are well-characterized and described, the protein components of most are not known.

**Figure 1:**
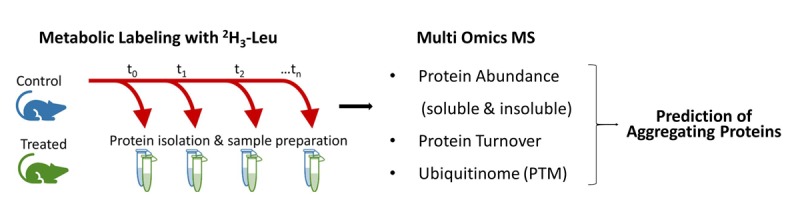
**Experimental design and workflow for predicting aggregating proteins *in vivo* in mice [****2****].** Following a treatment period, mice are metabolically labeled with ^2^H_3_-Leu over a specified timeframe (e.g. 17 days) to allow the calculation of protein turnover. Tissues are collected from mice in all experimental groups over multiple timepoints and processed into three fractions: ubiquitinated proteins, insoluble proteins, and soluble proteins. The ubiquitinated protein fraction is prepared via antibody enrichment. A multi-omic analysis that combines information about protein ubiquitination state, abundance in either the soluble or insoluble fractions, and turnover rate predicts proteins which are likely components of insoluble protein aggregates.

A common feature of protein aggregates is extensive post-translational modification of proteins with ubiquitin. Ubiquitination is commonly associated with the turnover of proteins. The ubiquitin-proteasome system, one of the major protein quality control pathways of the cell, uses poly-ubiquitination of proteins as a signal for protein degradation by the proteasome. Ubiquitination also promotes autophagic turnover pathways such as mitophagy and aggrephagy, a selective disposal of protein aggregates [1]. The ubiquitination of protein aggregates can act as a signal to degrade proteins through one of these mechanisms. However, the accumulation of ubiquitin-positive aggregates with aging represents a failure of protein turnover through these pathways. Hence, by measuring the rates of turnover of ubiquitinated proteins, we can determine the identity of long-lived ubiquitinated proteins (Figure 1) [2]. Immunofluorescence can subsequently be used to confirm the aggregation and localization of these proteins.

Proteomic methods for measuring rates of protein turnover *in vivo* have now been described in numerous organisms [1]. In mice, protein turnover can be determined by coupling metabolic labeling with mass spectrometry and examining the appearance or loss of a heavy isotopic label in peptides over time [1]. A critical factor in the calculation of protein turnover following metabolic labeling is determining isotopic enrichment in the amino-acid precursor pool from which new proteins are synthesized. Recently developed software tools, such as Topograph [3], enable the determination of precursor enrichment based on the relative intensity of chromatographic peaks from peptides containing two or more heavy isotope-labeled amino acids. These tools have been used to determine the turnover rates of thousands of proteins during aging. A recent study reported protein turnover rates in several tissues in young and aged mice fed *ad libitum*, calorie restricted, or treated with rapamycin [4]. The authors found that global protein turnover was significantly altered during aging, and many of these changes were reversed after 8 weeks of either calorie restriction or rapamycin treatment [2,4]. Along with changes in turnover with aging, the authors observed decreases in protein quality and increased protein ubiquitination that were also reversed by treatments.

To examine the link between increased ubiquitination and altered protein turnover, Basisty et al. developed a method to measure the turnover of ubiquitinated proteins [2]. Following metabolic labeling, ubiquitinated proteins were enriched from mouse livers using bead-antibody conjugates. This large-scale, high-throughput strategy for proteomic assessment of ubiquitinated proteins and specifically the combination with measurements of protein turnover [2] opens new opportunities to investigate aging organisms or age-related disease models.

A key consideration in the determination of ubiquitinated protein turnover is combining a stable isotope- heavy leucine -feeding study with PTM enrichment and mass spectrometric analysis and specialized software tools and data processing to perform the calculation of ubiquitinated protein turnover [2].

In aged mice there is a striking reduction in protein turnover and an increase in abundance of insoluble ubiquitin-modified proteins compared to young mice. This accumulation of ubiquitinated proteins suggests that old mice lack the proteostatic capacity to eliminate ubiquitinated proteins. Moreover, calorie restriction and rapamycin treatment, interventions known to enhance proteostasis, restored the turnover of ubiquitinated proteins in old mice.

Given that protein aggregates are insoluble, often ubiquitinated, and difficult to degrade, it was reasoned that the slowly turned-over ubiquitinated proteins in less soluble protein fractions that accumulated with age may be overrepresented in protein aggregates. To test this, two of the top “aggregator” protein candidates, keratin 8 and catalase, were measured by immunofluorescence. Both keratin 8, known to be the primary component of liver aggregates known as ‘Mallory Bodies’, and catalase, a previously unknown component of protein aggregates, were confirmed. These findings suggest that the inability of old mice to turn over ubiquitinated proteins is in part due to their sequestration into insoluble protein aggregates.

This work demonstrates that using a multi-omic approach which integrates changes in relative protein abundances, rates of turnover, and modification of proteins may provide novel mechanistic insights into protein homeostasis, particularly in identifying novel aggregating proteins (Figure 1). This approach may also identify soluble and non-aggregating proteins which fail to turn over appropriately and presumably escape degradation through other mechanisms. Coupling enrichment of other post-translational modifications with the analysis of protein abundance and turnover may reveal similar insights into the behavior of other modified protein populations [5].

In future studies, it will be of considerable interest to perform similar analysis in other aged tissues, particularly in tissues with a susceptibility to the formation of protein aggregates and inclusions, such as the brain, heart, and eye. Identifying the protein components of insoluble age-related aggregates will provide mechanistic insights into the formation of aggregates and new possible targets for therapeutic interventions in age-related diseases.
